# Huge buccal angiomyolipoma: a rare entity^☆^

**DOI:** 10.1016/j.bjorl.2017.08.002

**Published:** 2017-08-24

**Authors:** Siew Chung Cheah, Rohaizam Jaafar, Murni Hartini Jais

**Affiliations:** a Miri General Hospital, Department of Otorhinolaryngology-Head & Neck Surgery, Sarawak, Malaysia; b Sarawak General Hospital, Department of Pathology, Sarawak, Malaysia

## Introduction

Angiomyolipoma is a benign tumour composed of mature fat cell, thick-walled blood vessels and smooth muscle cells.^1^ It commonly involves the kidney and about 50% of patients with renal angiomyolipoma also have tuberous sclerosis complex.^2^ Many cases of extrarenal angiomyolipomas have been reported in the past, liver being the most common site. Oral region involvement is extremely rare. To date, only 1 case of angiomyolipoma of buccal mucosa has been reported.^3^

## Case report

A 28 year-old gentleman presented with gradually enlarging, painless left buccal swelling for 1 year duration. There was no history of bleeding from the swelling and no history of previous dental treatment. On further questioning, there was no significant past medical and surgical history. He is a smoker with 2.5 pack years and social drinker. Examination revealed a well circumscribed swelling over left buccal mucosa approximating 3.0 × 2.0 cm. The mass was firm, non-tender and mobile, covered by normal mucosa. It was not attached to the bone or mucosa on bimanual examination. No other swelling over the left extra oral region was found. He had no family history of tuberous sclerosis and no cutaneous signs of tuberous sclerosis.

**Figure 1:**
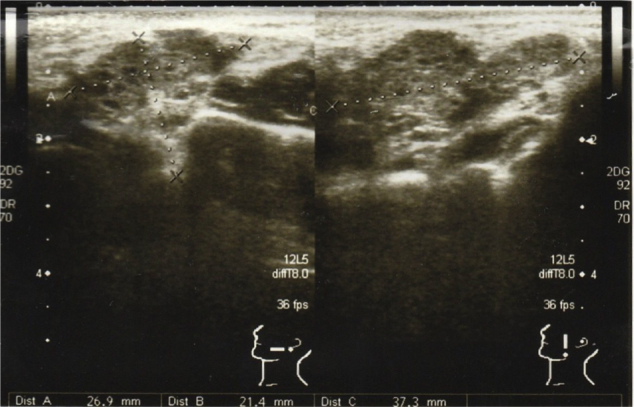
Ultrasound of left cheek showed a mass of 26.9 × 21.4 × 37.3 mm.

**Figure 2:**
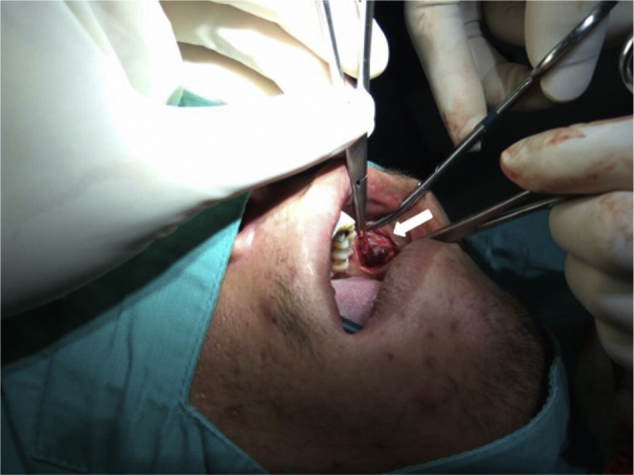
Intraoral excision of the left buccal swelling (arrow).

**Figure 3:**
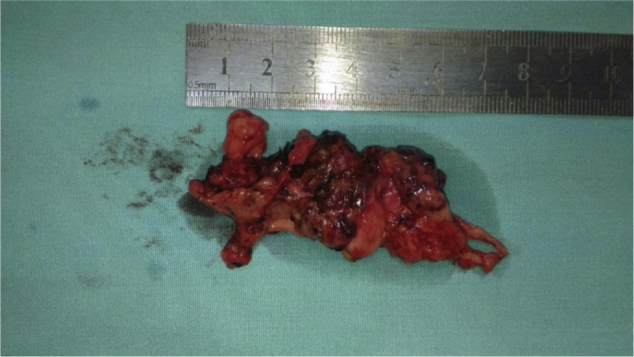
The gross specimen showed multilobated, black bluish in colour measured 60.0 × 33.0 × 20.0 mm.

**Figure 4:**
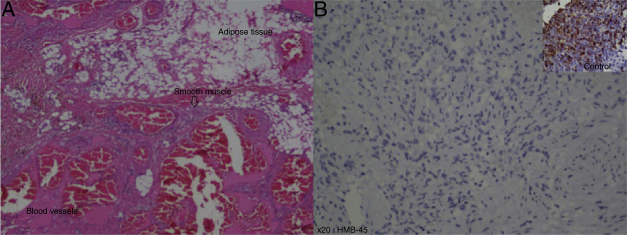
(A) Histological examination showed presence of mature adipose tissue, smooth muscle and thick walled blood vessels component (haematoxylin and eosin, original magnification 20×); (B) immunohistochemistry examination of smooth muscle with HMB-45 showed negative result, compared to the control over right upper corner (original magnification 20×).

**Figure 5:**
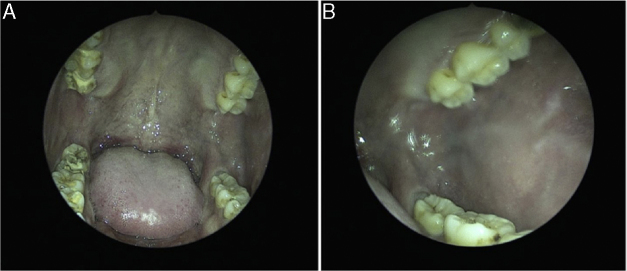
(A) The oral cavity 1 month after excision; (B) the left buccal mucosa 1 month after excision.

Ultrasound scan revealed a left cheek subcutaneous solid mass suspicious of neoplastic lesion measuring 26.9 × 21.4 × 37.3 mm (Fig. 1). A fine-needle biopsy was performed thereafter with diagnosis of infected cyst content. In view of suspicious radiological findings, an intraoral excision of the swelling was done under local anaesthesia using Scandonest 2% L (Mepivacaine hydrochloride 2% with Levonordefrin 1:20 000) after the informed consent from patient (Fig. 2). The specimen was multilobulated, black bluish in colour, measured about 60.0 × 33.0 × 20.0 mm (Fig. 3). Histopathologic examination of the tumour revealed a mixture of predominant mature fat and thick walled poorly organized blood vessels. Some spindle smooth muscle cells appeared to emanate from blood vessel walls in radial pattern with absence of epithelioid smooth muscle cell. Immunohistochemistry examination showed that the smooth muscle cells within the tumour were negative for HMB-45 (Fig. 4). Two week post-operatively, the wound healed without any recurrence of swelling noted (Fig. 5).

## Discussion

Angiomyolipoma is rare tumour of the oral cavity, and exceptionally rare in the buccal mucosa.^1^ There were only 17 cases of angiomyolipoma in English published work.^2^, ^3^, ^4^, ^5^, ^6^, ^7^, ^8^, ^9^ There are many different locations, including hard palate, upper lip, lower lip, tongue, base of tongue and buccal mucosa. Only 1 out of 17 cases of angiomyolipoma was found in the buccal mucosa.^3^ The mean size of angiomyolipomas in the oral region was smaller than 40.0 mm, except one case in the tongue which was 80.0 mm in diameter.^4^ In our current case, the angiomyolipoma was 60.0 × 33.0 × 20.0 mm, the largest found in buccal mucosa.

The age of occurrence varies from second to eighth decades with no sex prevalence.^2^, ^5^, ^6^ Only one case was described to have association with tuberous sclerosis complex previously.^3^ Extrarenal angiofibromas are rarely associated with tuberous sclerosis complex.^2^, ^9^ Genetic studies were not done in our patient because it was not available in our centre. There was, however, no past medical history of seizure, family history of tuberous sclerosis complex and other cutaneous lesions found in tuberous sclerosis complex. Undiagnosed disease may lead to complications such as obstructive nephropathy, hydrocephalus and pulmonary hypertension. In the head and neck region, the tumour may cause trismus, difficulty in swallowing and facial disfigurement if left untreated.

Microscopically, renal and liver angiomyolipomas showed distinct features from other locations because the presence of epithelioid cell.^2^, ^5^, ^6^, ^7^, ^8^, ^9^ The term ‘mucocutaneous angiomyolipoma’ was proposed previously in a report of nasal mucosa angiomyolipoma. Angiomyolipomas that arise from oral cavity, nasal cavity and skin were grouped under mucocutaneous angiomyolipoma because of their similarity in microscopic features.^2^, ^7^ HMB-45 is positive in tumours associated with tuberous sclerosis complex. Angiomyolipomas found in the head and neck region were different from those in renal and liver because of negativity in immunohistochemical staining with HMB-45.^5^, ^6^ As in our case, the microscopic examination showed absence of epithelioid cell and also negative staining for HMB-45 for smooth muscle cell. Recurrence was not reported so far. Hence, complete excision is considered curative.^5^, ^7^, ^9^

Preoperative diagnosis was a challenge. As in our patient, initial radiological investigation by ultrasound showed a solid mass suspicious of sarcoma. Fine needle aspiration for cytology yielded only infected cyst content. Finally, an excisional biopsy was performed to confirm the diagnosis. The differential diagnosis in this case includes haemangioma and angiolipoma because the gross specimen is black bluish in colour. Although fat and blood vessels components are greater than smooth muscles, the presence of smooth muscle bundles under microscopic examination is quite significant which lead to the final diagnosis of angiomyolipoma.

We decided to perform intraoral excision under local anaesthesia because there was no bone or muscle infiltrations from the ultrasound scan which may complicate the operation. Moreover, local anesthetic excision involved a shorter waiting time than surgery performed under general anaesthesia. In summary, we present a case of angiomyolipoma which is the largest reported among those in buccal mucosa. Complete excision is important for both diagnostic and curative purpose.

## Conclusion

Angiomyolipoma of the oral cavity is very rare especially in buccal mucosa. Angiomyolipoma should be considered in the differential diagnosis of cheek swelling apart from hemangioma and angiolipoma. Hence, a complete excision is important for both diagnostic and curative purpose.

## Conflicts of interest

The authors declare no conflicts of interest.
